# Genome-wide profiling identifies the genetic dependencies of cell death following EGFR inhibition

**DOI:** 10.1016/j.jbc.2026.111414

**Published:** 2026-04-02

**Authors:** Sydney A. Porto, Gavin A. Birdsall, Nicholas W. Harper, Megan E. Honeywell, Scott M. Leighow, Tiana E. Naylor, Kelly M. Ward, Mika K. Wesley, Justin R. Pritchard, Michael J. Lee

**Affiliations:** 1 Department of Systems Biology, UMass Chan Medical School, Worcester, Massachusetts, USA; 2 Department of Biomedical Engineering, and Huck Institute for The Life Sciences, The Pennsylvania State University, University Park, Pennsylvania, USA

**Keywords:** cancer therapy, cell death, drug action, epidermal growth factor receptor (EGFR), functional genomics, lung cancer, systems biology

## Abstract

EGFR is a proto-oncogene that is mutationally activated in a variety of cancers. Small molecule inhibitors targeting EGFR can effectively slow the progression of disease, and in some settings, these drugs even cause dramatic tumor regression. However, responses to EGFR inhibitors are rarely durable, and the mechanisms contributing to response variation remain unclear. In particular, several distinct mechanisms have been proposed to explain how EGFR inhibition activates cell death, and a consensus has yet to emerge. In this study, we use functional genomics with specialized analyses to infer how genetic perturbations affect the drug-induced death rate. Our data clarify that inhibition of PI3K signaling drives the lethality of EGFR inhibition. Inhibition of other pathways downstream of EGFR, including the RAS-MAPK pathway, promotes growth suppression but not the lethal effects of EGFR inhibitors. Taken together, our study provides a “reference map” for the genome-wide genetic dependencies of lethality in response to EGFR inhibitors.

Molecularly targeted therapies have revolutionized cancer treatment. Targeted therapies aim to distinguish between normal cells and cancer cells, ideally leading to highly selective killing of cancer cells with low toxicity to healthy normal tissue. These treatments are typically designed to inactivate oncogenes in growth factor signaling pathways. While these treatments have been successful in many settings, responses are generally not durable (1). Previous research on the failures of targeted therapies has focused on understanding the mechanisms of drug resistance (2). However, less attention has been devoted to determining the mechanisms of drug sensitivity and, in particular, why inhibition of a growth factor oncogene activates tumor cell death in the first place. The term “oncogene addiction” was coined to describe this phenomenon, highlighting that cell death in this context is an atypical response observed only in some cancers (3, 4). However, in the decades since oncogene addiction was first described, we still have a limited understanding of why cell death is activated following loss of growth factor signaling and why this is only observed in some cancers.

Some of the earliest and most well-studied molecularly targeted therapies are tyrosine kinase inhibitors targeting the epidermal growth factor receptor (EGFR). First-generation EGFR inhibitors, including gefitinib and erlotinib, reversibly bind to EGFR and inhibit the binding of ATP, consequently abolishing the receptor's tyrosine-kinase activity (5). These two drugs had remarkable success in patients with EGFR-mutant non-small cell lung cancer (NSCLC) (6, 7, 8), and erlotinib was ultimately approved as a first-line treatment for NSCLC patients harboring an EGFR exon 19 deletion or exon 21 L858R substitution mutation (9). However, the success of first-generation EGFR inhibitors was limited due to the emergence of drug-resistant populations, including the EGFR T790M “gatekeeper” mutation (10, 11, 12). Second- and third-generation EGFR inhibitors, such as osimertinib, were developed to address this issue of resistance, although novel resistance mutations continued to arise (13).

In cases where EGFR inhibitors are effective treatment strategies, it remains unclear why inhibiting EGFR activates cell death. Several major signaling pathways downstream of EGFR control cell proliferation and survival, most notably the RAS-RAF-MEK-ERK and PI3K-AKT-mTOR pathways (14). Previous studies have suggested that EGFR inhibition might cause cell death through either the RAS pathway, the PI3K pathway, a combination of both pathways, or another unique downstream pathway (15, 16, 17). Given this ambiguity, there remains an unmet need to better define how EGFR inhibitors cause cell death. A detailed understanding of EGFR inhibitor-induced lethality mechanisms may aid in identifying patients likely to respond well to these drugs and help predict novel resistance mechanisms or more effective drug combinations.

Functional genomics can be used to identify the genetic dependencies of drug sensitivity, and theoretically, also the mechanisms of drug-induced cell death. CRISPR/Cas9-mediated genome editing can be used to functionally characterize all genes and/or gene regulatory elements (18). When conducted in the context of a drug treatment, these data are often referred to as a “chemo-genetic profile” (19). Chemo-genetic profiling has been effective at characterizing the genetic dependencies for “cell fitness.” However, these chemo-genetic profiles are not consistently able to identify mechanisms of drug-induced lethality. The central issue is that growth rate variation among knockout clones changes the relationship between population size and the rate of drug-induced cell death. These features confound the interpretation of chemo-genetic profiling data, often masking the degree to which genetic perturbations alter cell death. To address this issue, we recently developed a Method for Evaluating Death Using a Simulation-assisted Approach (MEDUSA) (20, 21). MEDUSA employs computational simulations that model clonal dynamics to reveal the combination of growth and death rates that created an observed drug response, and how each single gene knockout alters these rates.

In this study, we examined the response of EGFR-mutant NSCLC cells to the first-generation EGFR inhibitor, erlotinib, and the third-generation inhibitor, osimertinib. We determined that, although these drugs consistently induce growth inhibition, cell death is variable and dependent on the cell type/inhibitor combination. This is true even among NSCLC cell lines harboring the same EGFR mutations. Drug-induced changes in gene expression could not account for the observed differences in lethality across genetic backgrounds. Using chemo-genetic profiling with a MEDUSA-based analysis, we uncovered the genetic determinants of cell death following EGFR inhibition. MEDUSA highlights that the lethality of EGFR inhibitors depends on their ability to inhibit the PI3K signaling pathway. Notably, the insights generated by MEDUSA were distinct from insights generated using conventional chemo-genetic profile analyses. Taken together, these data provide a comprehensive reference map for the genetic dependencies underlying EGFR inhibitor-induced cell death.

## Results

### EGFR inhibitors rarely activate cell death while consistently promoting growth inhibition in EGFR-mutant NSCLC

**Figure 1:**
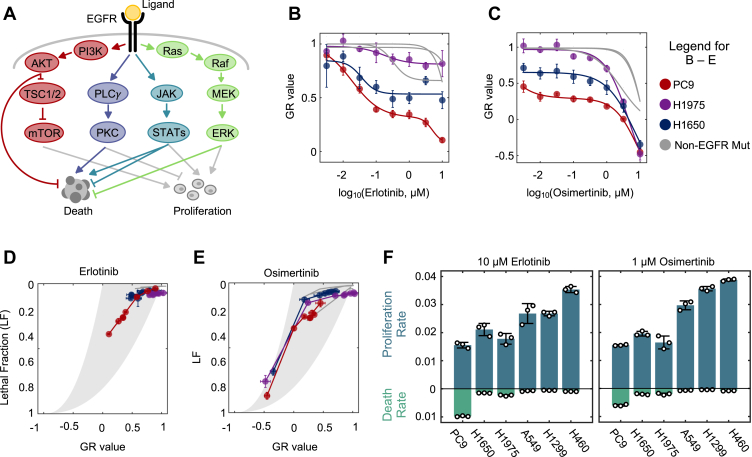
**EGFR inhibitors rarely activate cell death, even in EGFR-mutant NSCLC cells.***A*, simplified schematic of key signaling pathways that control survival and proliferation downstream of EGFR. *B*, erlotinib sensitivity of PC9, H1975, H1650, and three non-EGFR mutant NSCLC cells, evaluated 72 h following erlotinib exposure. GR value reports the net population growth/shrinkage rate, relative to untreated cells. *C*, as in (*B*) but following exposure to osimertinib. *D*, drug GRADE analysis for following 72-h exposure to erlotinib. Cell lines are colored as in panel (*B*) and (*C*). GRADE analysis juxtaposes the GR value with the drug-induced Lethal Fraction (LF) to visualize the drug-induced coordination between growth and death. Each cell line was exposed to erlotinib at an eight-point dose titration, as in panels (*B*) and (*C*). *E*, as in panel (*D*) but following exposure to osimertinib. *F*, proliferation rate (doublings/hour) and death rate (LF/hour) for cell lines treated with 10 μM erlotinib or 1 μM osimertinib. Values calculated from GRADE analysis. For all panels with error bars, data are the mean ± S.D. for *n =* 3 independent biological replicates.

EGFR signals through several downstream pathways that regulate cell survival and proliferation. Four of these canonical pathways are: the RAS-RAF-MEK-ERK, PI3K-AKT-mTOR, PLC *γ*, and JAK-STAT pathways (Fig. 1*A*) (14, 22). Prior studies have specifically connected EGFR inhibitor-driven cell death to the inhibition of either one or multiple pathways, although a consensus model has yet to emerge. For instance, several studies have suggested that EGFR inhibitor-induced cell death is driven by the inhibition of the RAS-RAF-MEK-ERK pathway, resulting from the loss of ERK-mediated BIM phosphorylation (23, 24, 25). BIM is a pro-apoptotic protein whose degradation is regulated by ERK, such that upon ERK inhibition, BIM protein accumulates and may activate apoptosis (15). In contrast, other studies suggest that the dual inhibition of both the RAS-RAF-MEK-ERK and PI3K-AKT-mTOR pathways drives apoptosis, with inhibition of the former leading to the upregulation of BIM, and inhibition of the latter leading to the downregulation of the anti-apoptotic protein Mcl-1 (16). Other pathways downstream of EGFR have also been linked to cell death. For instance, nuclear import of PKCδ has been shown to activate apoptosis (26, 27). Furthermore, STAT1 and STAT3 have both pro- and anti-apoptotic functions (28, 29). Given these numerous and conflicting mechanisms that describe how pathways downstream of EGFR contribute to cell death activation, the mechanisms of drug sensitivity following EGFR inhibition remain unclear.

To evaluate the mechanisms of lethality following EGFR inhibition, we began by profiling EGFR inhibitor sensitivity in a panel of EGFR-mutant and EGFR-wild-type NSCLC cell lines. We focused on the first-generation inhibitor, erlotinib, and the third-generation inhibitor, osimertinib. Because we tested these drugs across cells with varied growth rates, drug sensitivities were evaluated using the normalized growth rate inhibition (GR) value (Fig. S1*A*) (30). GR values account for growth rate variation between cells, therefore allowing for more accurate comparisons of drug responses across contexts. GR values are scaled between one and -1, where a GR value between 0 and one reports an expanding population, a value of 0 represents complete cytostasis, and a value between 0 and −1 indicates a shrinking population.

Previous studies have generally suggested that EGFR wild-type NSCLCs are less sensitive to EGFR inhibitors than NSCLCs containing activating mutations in EGFR (31). Consistent with these prior results, we find that EGFR mutant cell line PC9 (EGFR *delE746-A750*) had the highest sensitivity to erlotinib and osimertinib (Fig. 1, *B* and *C*). However, other EGFR mutant NSCLC cells, H1650 (*delE746-A570*), and H1975 (L858R, T790M), were only modestly more sensitive than the EGFR wild-type NSCLCs that we profiled (Figs. 1, *B* and *C*, and S1, *B* and *C*). These results were consistent with prior studies, which reported that PC9 cells are particularly sensitive to EGFR inhibition (32).

We next sought to further characterize the increased sensitivity to EGFR inhibitors in PC9 cells. The GR value reports the net population growth rate at each dose of drug; however, GR values alone cannot clarify the relative contributions of growth inhibition *versus* cell death activation to the observed drug response. To determine the proportional contributions of growth inhibition and cell death activation following exposure to EGFR inhibitors, we used the Growth Rate Adjusted for DEath (GRADE) analysis approach (Fig. 1, *D* and *E*) (33). The GRADE method juxtaposes the GR value (*i.e.*, the net population growth rate) and the drug-induced lethal fraction (LF; *i.e.*, the average death rate). This analysis method enables a visualization of how growth inhibition and death activation are coordinated by a given drug. Furthermore, the data from GRADE analysis can also be used to infer the quantitative combinations of growth rates and death rates that generated the observed drug responses for each tested cell type (34).

GRADE analysis revealed that PC9 cells respond to erlotinib and osimertinib with a combination of reduced proliferation and high levels of cell death activation (Fig. 1, *D* and *E*). For instance, at a 10 μM dose of erlotinib, quantitative inference from GRADE analysis revealed that PC9 cells proliferate at 0.015 population doublings per hour, roughly half of their proliferation rate in untreated conditions, while also dying at 0.01 percent per hour, roughly 10 times their basal death rate (Fig. 1*F*). In all other contexts, EGFR inhibition only reduced the proliferation rate of these cells to ∼60 to 90% of the untreated growth rate, without causing significant levels of drug-induced death (Figs. 1*F* and S2, *A* and *B*). A notable exception was observed for high doses of osimertinib, which appear to kill all cells, likely due to off-target effects at these concentrations (Figs. 1*E* and S2*B*). Therefore, we found that EGFR inhibitors cause variable responses across the cell lines included in this panel, even among the three EGFR-mutant NSCLCs tested. With respect to the six cell lines used in this study, erlotinib and osimertinib appear to be uniquely lethal in PC9 cells.

### Drug-induced changes in gene expression do not account for the variable lethality observed following exposure to EGFR inhibitors

**Figure 2:**
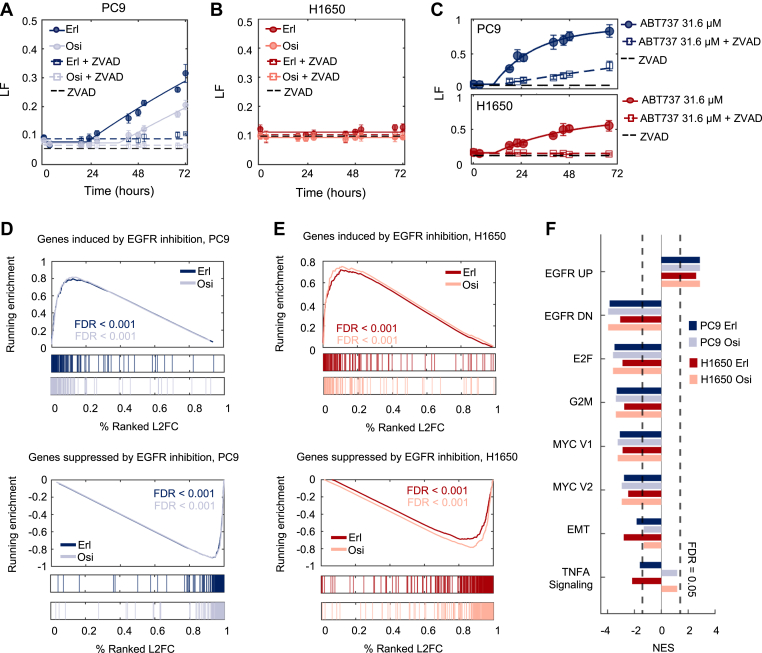
**Drug-induced changes in gene expression cannot explain the observed variation in the lethality of EGFR inhibitors.***A*, lethal fraction (LF) kinetics in PC9 cells, measured using the FLICK assay following exposure to either 10 μM erlotinib or 0.1 μM osimertinib, in the presence or absence of 50 μM ZVAD. *B*, as in panel (*A*) but in H1650 cells. *C*, cell death kinetics in PC9 (*top*) or H1650 (*bottom*) following exposure to ABT737, in the presence or absence of 50 μM ZVAD. *D*, gene set enrichment analysis (GSEA) for erlotinib- and osimertinib-induced gene expression changes in PC9 cells. Gene signatures used are from msigDB (“Kobayashi EGFR Signaling 24-h UP” and “Kobayashi EGFR Signaling 24-h DN”). FDR-adjusted *p*-values are based on 1000 permutations of gene sets. *E*, as in panel (*D*) but for H1650 cells. *F*, GSEA for drug-treated conditions compared to untreated samples for PC9 and H1650. Significantly changed enriched Hallmark Gene sets shown. For all panels with error bars, data are the mean ± S.D. for *n =* 3 independent biological replicates.

In the panel of cells we profiled, elevated lethality following EGFR inhibition was observed only in PC9 cells. Therefore, we aimed to further characterize why EGFR inhibition was more effective at promoting cell death in this context. To address this, we examined the kinetics of EGFR inhibitor-induced death in PC9 and H1650, two cell lines harboring the same EGFR mutation (*delE746-A750*), which exhibit different levels of sensitivity to EGFR inhibition (Fig. 1*F*). Kinetic analysis of cell death revealed that high-dose erlotinib (10 μM) kills ∼30% of PC9 cells over a 72-h exposure period (Fig. 2*A*). Osimertinib was significantly more potent, with a 100-fold lower dose (0.1 μM) resulting in a similar level of lethality (Fig. 2*A*). To explore the subtype of cell death activated by erlotinib or osimertinib in PC9 cells, we quantified the drug response in the presence and absence of z-VAD, an inhibitor of apoptotic caspases. For both erlotinib and osimertinib, z-VAD significantly inhibited EGFR inhibitor-induced lethality, and inhibitors of other cell death pathways did not significantly alter the level or the timing of cell death (Figs. 2*A* and S3*A*). These data suggest that EGFR inhibition activates an apoptotic cell death, which is consistent with prior studies (35, 36, 37). Importantly, kinetic evaluation of EGFR inhibition in H1650 cells revealed that the modest death rate observed is not drug-induced, but rather results from a high level of background death in these cells (Fig. 2*B*). Both PC9 and H1650 cells were similarly sensitive to other apoptotic agents, such as the BH3 mimetic, ABT737, the proteasome inhibitor, Bortezomib, or the Pol II degrader, Triptolide (Figs. 2*C* and S3*B*). Thus, PC9 cells experience drug-induced apoptotic lethality when exposed to EGFR inhibition, while H1650 cells do not.

Given that PC9 and H1650 cells have such different responses to EGFR inhibition, we next aimed to determine if the lethality observed in PC9—or the lack of lethality in H1650—could be explained by differences in drug-induced gene expression changes. To test this, we performed RNA-sequencing in PC9 and H1650 cells 36 h after exposure to erlotinib or osimertinib (the time of death onset in PC9 cells) (Fig. S3, *C*–*F*). We used gene set enrichment analysis (GSEA) to quantify and visualize the drug-induced expression profiles (38). Erlotinib- and osimertinib-induced changes in gene expression are well characterized; therefore, we first compared our results with previously annotated genetic signatures for the transcriptional effects of EGFR inhibition in EGFR-mutant NSCLC. For instance, Kobayashi *et al.* previously characterized gene expression signatures comprised of 102 or 253 genes that were up- or downregulated, respectively, specifically in NSCLC cells sensitive to EGFR inhibition but not in insensitive cells (39). In PC9 cells exposed to either erlotinib or osimertinib, the transcriptional effects that we observed were similar to those observed previously in EGFR-mutant NSCLCs that are sensitive to EGFR inhibition (Fig. 2*D*). Surprisingly, however, nearly identical gene expression changes were also observed following erlotinib and osimertinib exposure in H1650 cells, despite the relative lack of sensitivity and complete lack of drug-induced cell death that we observe following EGFR inhibition (Fig. 2*E*).

To explore the similarities and differences between the transcriptional responses in PC9 and H1650 more broadly, we also explored other gene signatures impacted by EGFR inhibition in these cells. Again, for both cell lines and both EGFR inhibitors, similar transcriptional responses were observed across all hallmark gene signatures. Overall, we failed to identify any differences in the transcriptional responses to EGFR inhibition that could clarify the observed differences in drug-induced lethality (Fig. 2*F* and Table S1). Furthermore, at the protein level, drug-induced changes in the expression of key apoptotic regulatory proteins were also similar in PC9 and H1650 cells. For instance, the pro-apoptotic protein, BIM, has been reported to increase in expression following EGFR inhibition (24). Indeed, we observed EGFR inhibitor-induced upregulation of BIM at both the mRNA and protein levels (Fig. S3, *G* and *H* and Table S1). However, these changes were similar in PC9 and H1650 cells, suggesting that BIM induction is not sufficient for driving EGFR inhibitor-induced lethality. Taken together, these data show that in EGFR inhibitor-sensitive PC9 cells and EGFR inhibitor-insensitive H1650 cells, EGFR inhibition has a similar effect on gene expression despite the significant differences in drug-induced lethality. Thus, drug-induced changes in gene expression appear not to account for the differences in lethality following EGFR inhibition.

### MEDUSA-based analysis reveals mechanisms of lethality following EGFR inhibition

**Figure 3:**
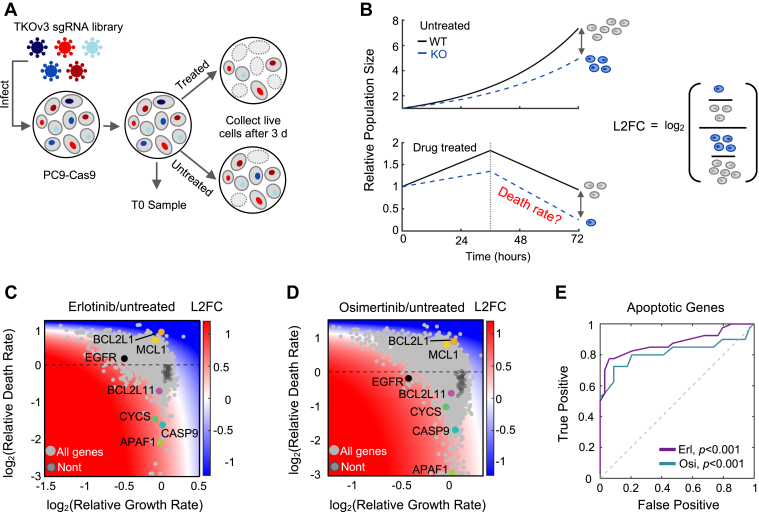
**Chemo-genetic profiling with MEDUSA-based analysis reveals the genetic dependencies of lethality following EGFR inhibition.***A*, schematic of chemo-genetic profiling using a pooled sgRNA library. *B*, model showing the relationship between drug-induced growth and death rates, and the conventional chemo-genetic profiling analysis (log2-Fold Change, L2FC). *C*, gene-level chemo-genetic profiling with MEDUSA analysis for PC9 cells treated with 10 μM erlotinib for 72 h. Non-targeting genes are shown in *dark gray*. Several known apoptotic regulators and EGFR are highlighted. *D*, as in panel (*C*) but following 0.1 μM osimertinib exposure. *E*, ROC analysis for MEDUSA-inferred death rates of known apoptotic genes. Empiric *p* value based on AUC and bootstrapping with 1000 iterations.

Since changes in gene expression were unable to explain the mechanisms of EGFR-inhibitor-driven death in PC9 cells, we sought to clarify the mechanisms of lethality by identifying the genetic dependencies of EGFR-inhibitor-induced cell death in PC9 cells. We performed a genome-wide single gene knockout screen using the TKOv3 library (Figs. 3*A* and S4*A*) (40). Cells were treated with either 10 μM erlotinib or 0.1 μM osimertinib for 72 h, which resulted in ∼30 to 40% of the population dying (Fig. 2*A*). To examine the quality of our chemo-genetic profiling data, we first explored the degree of correlation between our biological replicates (41). These data revealed a high correlation between replicates, both at the level of sequencing read counts and at the level of drug-induced fold change, suggesting a high degree of biological reproducibility (Counts: r = 0.97; Fold change: r = 0.82; Fig. S4, *B* and *C*). Furthermore, when comparing the “T0” input samples to the untreated samples at the end of the assay, we observed that core essential genes dropped out over time, suggesting that our genetic profiling data accurately recovered expected genetic dependencies (Fig. S4*D*).

To identify genes that regulate EGFR inhibitor-induced cell death, we employed the recently developed MEDUSA algorithm (20). Conventional chemo-genetic profiles rely on examining the relative abundance of each clone, comparing treated and untreated populations (Fig. 3*B*). These methods—which are effectively comparing four dynamic populations, each with its own growth and death rates—fail to capture how genetic perturbations affect drug-induced cell death, due to the confounding effects of variable proliferation rates and death rates within the other populations (Fig. 3*B*) (20). The MEDUSA method solves this issue using comprehensive simulations of the drug-treated and untreated populations growing and dying at varied rates, to map the observed fold change data to a single pair of growth and death rates (see Experimental procedures) (20, 21). The outcome of a MEDUSA-based analysis is a computational inference of the drug-induced growth and death rates for each knockout clone in a chemo-genetic profiling dataset.

To evaluate the accuracy of our analysis, we began by determining MEDUSA's effectiveness at identifying genes involved in intrinsic apoptosis, as apoptotic regulation is reasonably well-studied. For instance, knocking out any component of the apoptosome—an essential apoptotic effector complex that is comprised of caspase-9 (*CASP9*), cytochrome c (*CYCS*), and APAF-1 (*APAF1*)—should compromise the drug-induced death rate for any drug that kills using apoptosis (42). As expected, our MEDUSA-based analysis revealed that knocking out *CASP9*, *CYCS*, or *APAF1* significantly suppressed the rate of erlotinib- or osimertinib-induced death (Fig. 3, *C* and *D*). MEDUSA also identified potent negative regulators of apoptosis, such as Bcl-xL (*BCL2L1*) and Mcl-1 (*MCL1*), which, when knocked out, sensitized cells to the lethal effects of both erlotinib and osimertinib (Fig. 3, *C* and *D*). Consistent with prior literature, which has highlighted the central importance of the pro-apoptotic protein BIM (*BCL2L11*) (23, 24, 25), knocking out BIM suppressed erlotinib-induced cell death, although the effect was somewhat modest (Fig. 3*C*). BIM knockout did not, however, significantly decrease drug-induced lethality in the context of osimertinib (Fig. 3*D*). Overall, MEDUSA-based analysis was effective at recovering known apoptotic regulatory genes (Fig. 3*E*). In contrast, a conventional “log-fold change” based analysis fails to recover some members of the apoptosome and other critical regulators of apoptosis (Fig. S4*E*).

Quantitatively, our chemo-genetic profiling data for erlotinib- or osimertinib-induced death were strongly correlated, suggesting an overall similarity between the mechanisms of lethality for these two drugs (r = 0.71; Fig. S4*F*). In total, our analysis identified 676 genes that significantly modulate the death rate in response to erlotinib treatment, as well as 674 genes that modulate death in response to osimertinib (Table S2). Of these, 359 genes were shared as regulators of both drugs. Taken together, these data represent the first consensus map of the genetic dependencies of lethality following EGFR inhibition in EGFR-mutant PC9 cells.

### Inhibition of PI3K signaling drives lethality following EGFR inhibition

**Figure 4:**
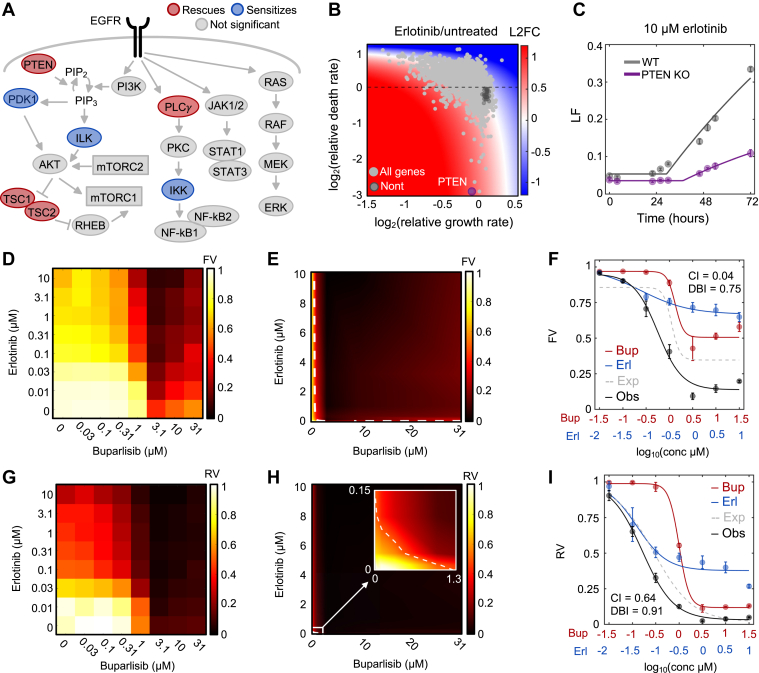
**Inhibition of PI3K signaling drives the lethality of EGFR inhibition.***A*, schematic of signaling pathways downstream of EGFR. Death regulatory genes identified in MEDUSA are highlighted. Genes whose knockout sensitizes death are shown in *blue*, while genes whose knockout rescues death are shown in *red*. *B*, Gene-level chemo-genetic profile for PC9 cells treated with erlotinib. *PTEN*, the top hit based on the MEDUSA death rate, is highlighted. *C*, validation of MEDUSA-inferred death rate for *PTEN*. Erlotinib-induced death evaluated using the FLICK assay. *D*, dose titration of erlotinib and buparlisib in PC9 cells. Heatmap is scaled by the mean fractional viability of three biological replicates following 72 h of drug treatment. *E*, isobologram analysis for the data in (*D*). The *dashed line* represents the erlotinib and buparlisib combinations that result in 50% response (50% isobol). *F*, dose curve for erlotinib and buparlisib at fixed ratio dosing. The expected dose curve in the case of additivity is shown in addition to the observed combination. The Chou-Talalay Combination Index (CI) and the Deviation from Bliss Independence (DBI) are shown. *G–I*, as in (*D–F*), but data are the conventional relative viability metric, rather than a death-specific metric. For all panels with error bars, data are the mean ± S.D. for *n =* 3 independent biological replicates.

Having determined that our screen successfully identified apoptotic regulatory genes, we next sought to use the genetic dependency data to identify which signaling pathways contribute to death following EGFR inhibition. We began by mapping the genes that regulate the lethality of EGFR inhibitors onto the canonical signaling pathways downstream of EGFR (Fig. 4*A*). We saw that knocking out several components of the PI3K pathway altered the erlotinib-induced death rate. Indeed, knocking out activators in the PI3K pathway, including PDK1 and ILK, sensitized PC9 cells to death (Fig. 4*A* and Table S2). Likewise, knocking out inhibitors of the PI3K pathway, including PTEN and both members of the TSC complex, rescued EGFR inhibitor-induced cell death (Figs. 4*A* and S5, *A* and *B*). The involvement of PLCγ signaling was less clear. Knocking out PLCγ (PLCG1) partially rescued viability; however, knocking out activators of this pathway did not consistently rescue viability (Table S2). Knocking out genes within the JAK/STAT and RAS/MAPK pathways did not alter EGFR inhibitor-induced cell death (Table S2).

Among the canonical signaling pathways downstream of EGFR, death regulation appeared to be unique to the PI3K pathway (Fig. S5*C*). Individual genes within the PLC *γ*, JAK/STAT, and RAS pathways generally did not contribute to EGFR inhibition-induced cell death, and overall, genes within these pathways were not significantly enriched for regulators of erlotinib- or osimertinib-induced cell death (Fig. S5*C*). It is worth noting, however, that genetic redundancy, particularly within the RAS pathway, may lead to a gene within these pathways failing to score in a screen focused on single gene knockouts (43, 44).

To validate the functional significance of the PI3K pathway in promoting EGFR inhibitor-induced cell death, we initially focused on genetically validating single-gene knockouts identified by our MEDUSA-based analysis as regulators of the lethality of erlotinib and osimertinib. PTEN is a phosphatidylinositol-3,4,5-trisphosphate (PIP_3_) phosphatase, which is a potent negative regulator of PI3K signaling (45, 46). Our MEDUSA-based analysis revealed that *PTEN* knockout strongly suppressed the lethality induced by EGFR inhibitors (Fig. 4*B*). To validate this insight, we knocked out *PTEN* using targeted sgRNAs. These data confirm that knocking out *PTEN* potently suppressed erlotinib-induced cell death (Figs. 4*C* and S5*D*). Similarly, knocking out activators of PI3K signaling, such as PDK1 (*PDPK1* gene) or PI3K (*PIK3CA* gene) sensitized cells to the lethality of both erlotinib and osimertinib (Fig. S5*E*).

To further validate the importance of PI3K signaling for promoting the lethality of EGFR inhibitors, we next sought to quantify the extent to which chemically inhibiting PI3K signaling alters erlotinib-induced lethality. To address this question, we tested erlotinib in the presence and absence of the PI3K inhibitor buparlisib, evaluating each drug across a large dose titration at all pair-wise dosing combinations (Fig. 4*D*). We scored single-drug and combinatorial drug responses using the “fractional viability” metric (FV, the number of live cells divided by the total number of live and dead cells). FV differs from conventional response metrics, which compare treated and untreated populations, and importantly, FV is a death-specific measurement (47). Using this death-specific analysis, we found that erlotinib and buparlisib induce moderate lethality as single agents but that the lethality is exacerbated when these drugs are used in combination (Fig. 4*D*).

To characterize the pharmacological interaction between erlotinib and buparlisib in PC9 cells, we used well-validated measures of drug–drug interactions (*i.e.*, drug “synergy” or “antagonism”) (47, 48). We began by visually inspecting the combinatorial response data using the Loewe isobologram convention. In the Loewe model, one would expect similarly efficacious doses to produce a linear relationship (*i.e.*, linear “isobols”) if the two drugs integrate additively. However, combinations of erlotinib and buparlisib produced overwhelmingly concave isobols, indicating potent drug synergy (Fig. 4*E*). To quantify the degree of drug synergy, we used the Chou-Talalay Combination Index (CI), which revealed a CI of 0.04, equivalent to a 25-fold reduction of the dose required for 50% lethality when compared to a dose additivity model (Fig. 4*F*). Similarly, strong synergy was observed when these data were evaluated using the Bliss Independence reference model (DBI, Fig. 4*F*). Similar results were observed for combinations of erlotinib and AKT inhibitors, MK2206 or GSK690693 (Fig. S6).

To further validate our MEDUSA-based analysis, we next focused on whether the inhibition of PI3K signaling specifically promoted the lethality—and not the growth suppression—of EGFR inhibitors. To address this question, we re-analyzed our erlotinib-buparlisib drug combination data using the conventional “relative viability” (RV) metric (Fig. 4*G*). RV is based on the size of the drug-treated population, compared to an untreated control population. In contrast to FV, which directly scores drug-induced death, RV is sensitive to changes in proliferation but relatively insensitive to changes in cell death (20, 33). Quantitative evaluation of these data revealed a modest, and statistically insignificant, trend towards drug synergy, with the erlotinib-buparlisib combination resulting in a 50% reduction in RV at a dose within 2-fold of the expectation based on dose additivity (CI = 0.64, Fig. 4, *H* and *I*). Taken together, these data confirm our MEDUSA-based inferences and highlight that inhibition of the PI3K signaling pathway specifically drives the lethality of EGFR inhibition, with only a modest impact on EGFR inhibitor-induced growth suppression.

### Genetic dependencies of lethality for EGFR inhibition are distinct from the genetic dependencies of cell fitness or cell proliferation

**Figure 5:**
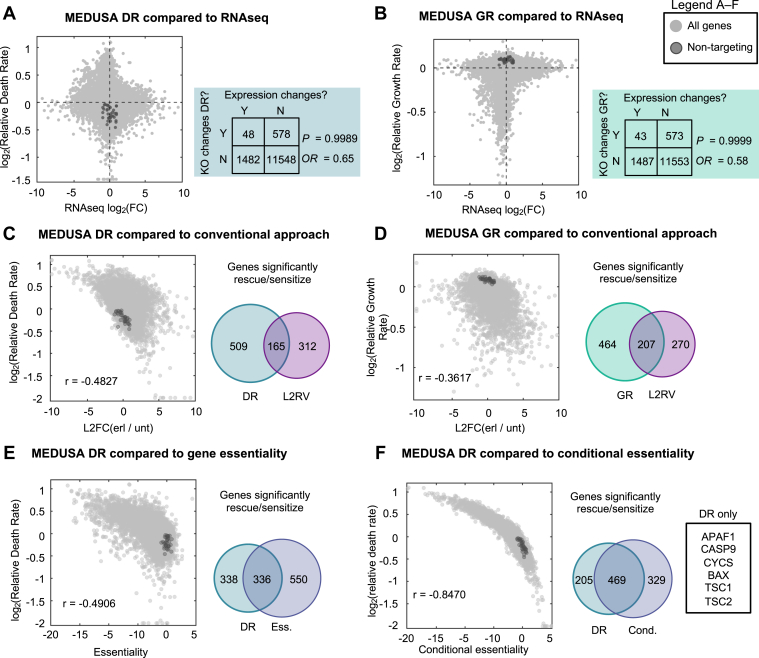
**Genetic dependencies of lethality are distinct from the genetic dependencies of cell fitness or cell proliferation.***A*, Comparison of MEDUSA-inferred death rate and drug-induced gene expression changes. *Shaded* regions of the graph genes that are significantly differentially expressed and whose deletion significantly alters the drug-induced death rate. Odds Ratio (OR) and *p*-value based on Fisher's Exact Test to determine the relationship between gene expression changes and death regulatory function. *B*, as in panel (*A*) but for the MEDUSA-inferred growth rate compared to differential gene expression. *C*, comparison of MEDUSA death rate to conventional chemo-genetic profiling analysis (L2FC). Venn diagrams show the relative number and relationship between “hits” recovered by each analysis strategy. *D*, as in *panel* (*C*) but for MEDUSA GR. *E* and *F*, further comparison of the MEDUSA DR metric to other conventional metrics used in functional genomics and/or chemo-genetic profiling. *E*, comparison between MEDUSA DR and gene essentiality. *F*, comparison between MEDUSA DR and conditional essentiality. Several genes only recovered by the MEDUSA analysis, but not conditional essentiality, are highlighted in (*F*).

Given that EGFR inhibitors are well-studied, we next determined whether the insights generated by our MEDUSA-based inference of death regulation are distinct from those generated using other methods. For instance, numerous studies have investigated the mechanisms regulating EGFR inhibitor efficacy by examining EGFR inhibitor-induced changes in gene expression (39, 49, 50). We identified more than 1400 genes that are differentially expressed following exposure to erlotinib; however, we observed no relationship between the differentially expressed genes and the ∼600 genes that regulate erlotinib-induced death (Fig. 5*A*). Similarly, drug-induced changes in gene expression were not correlated with the growth regulatory function of each gene (Fig. 5*B*). These observations mirror those made previously in the context of yeast stress responses, which also highlight that genes transcriptionally induced by a stress tend to be distinct from the genes that are functionally required for the stress response (51). Collectively, these observations demonstrate that the insights generated by gene expression-based analysis of EGFR inhibition are unrelated to the mechanisms that regulate drug-induced lethality.

In addition to the numerous studies that have used gene expression changes to infer mechanisms of drug action, several other studies have explored EGFR inhibitors using chemo-genetic profiling (52, 53, 54). Previous chemo-genetic profiling studies, however, have invariably used analysis methods that fail to accurately identify death regulatory genes (generally a log-fold change-based analysis, comparing the relative abundance of each knockout clone in drug-treated *versus* untreated conditions). We therefore compared our MEDUSA-inferred death rates to the genetic dependencies identified using a conventional log-fold change analysis (Log-2-fold change, L2FC). We observe that our data, re-analyzed using a conventional L2FC-based metric, are weakly correlated with the MEDUSA-based analysis, with a high degree of false-positive and false-negative inferences (Fig. 5*C*).

Notably, the MEDUSA-inferred growth rates for drug-treated cells were also not well-correlated with a conventional L2FC-based analysis; however, the MEDUSA-inferred growth rates were strongly correlated with measures of gene essentiality (*e.g.*, growth fitness when measuring endpoint *versus* initial “T0” input samples, r = .976) (Figs. 5*D*, and S6, *A* and *B*). The strong positive correlation between MEDUSA-inferred growth rates and gene essentiality scores further demonstrates the accuracy of MEDUSA-based growth and death rate inferences, suggesting that gene essentiality is generally a measure of proliferative fitness, rather than death regulation.

Conventional population fold-change-based analysis tends to benefit from lower drug dosing with population evolution over longer assay times. In contrast, MEDUSA-based analysis requires higher drug dosing, paired with relatively short assay times. Thus, the failure of conventional analyses to identify mechanisms of death regulation in our chemo-genetic profiling data could have stemmed from the fact that our experiments were explicitly optimized for analysis with MEDUSA. To address this issue, we also compared our MEDUSA-inferred genetic dependencies with chemogenetic profiling data that were experimentally collected in a manner that was optimized for analysis using more traditional approaches. As with our MEDUSA-based analysis, a conventional chemogenetic profile of osimertinib showed poor correlation with drug-induced changes in gene expression (Fig. S6*C*). Notably, the MEDUSA-inferred growth and death rates for each gene knockout were also poorly correlated with a chemo-genetic profile of osimertinib generated using a drug dose and assay length that was optimized for conventional analyses (Fig. S6, *D* and *E*). These data clarify that the MEDUSA-based analysis improves the accuracy and sensitivity of chemo-genetic profiling.

Although population fold-change-based methods, such as L2FC, are the most common methods used in chemo-genetic profiling studies, gene essentiality (*i.e.*, L2FC when comparing untreated endpoint and initial input “T0” samples) and/or conditional gene essentiality, (*i.e.*, L2FC when comparing drug-treated and T0 samples) can also be used to assess genetic dependencies (40). Similar to what was observed for conventional L2FC-based analysis of treated *versus* untreated conditions, we observed a weak negative correlation between the MEDUSA-inferred death rate and gene essentiality score, suggesting that essential genes tend to increase the erlotinib-induced death rate when knocked out (r = −0.49, Fig. 5*E*). However, despite the modest correlation between these measurements, gene essentiality was not an effective strategy for identifying mechanisms of drug-induced cell death, as the essentiality data was not effective at identifying an apoptotic mechanism of action for erlotinib.

Similar data were observed when comparing MEDUSA-inferred death rates and conditional gene essentiality, although the correlation between these two measures was very strong (r = −0.85, Fig. 5*F*). This is not surprising, given that conditional essentiality effectively scores the relative fitness in a drugged condition for each gene knockout compared to wild-type cells, which is a more biologically interpretable comparison that lacks the confounding effects inherent in a conventional fold-change-based analysis. Nonetheless, conditional essentially also failed to replicate the sensitivity and accuracy of MEDUSA-inferred death rates. For instance, MEDUSA alone identified key regulators of apoptosis, including all subunits of the apoptosome: APAF1, CASP9, and CYCS (Fig. 5*F*). Overall, as was true for all other analytical strategies, conditional essentiality failed to clearly identify an apoptotic mechanism of action for erlotinib. Taken together, these data demonstrate that MEDUSA-inferred growth and death rates provide quantitative insights that are not captured using other conventional analysis methods.

## Discussion

In this study, we explored the mechanisms by which EGFR inhibitors activate cell death in the context of EGFR-mutant NSCLC cells. Using death-specific assays—including MEDUSA, to identify death regulatory genes—our data highlight the central importance of inhibiting the PI3K signaling pathway. Inhibition of other signaling branches, including the RAS-MAPK pathway, inhibited the proliferation of PC9 cells without promoting high levels of cell death. Our data and analyses reveal that these phenotypes are challenging to interpret using conventional methods, including gene expression data, which has been widely used to infer mechanisms of drug function. Thus, the data and analytical strategies presented here represent a valuable resource for understanding how EGFR inhibitors activate cell death and how to potentiate these responses.

More generally, from the perspective of chemo-genetic profiling, our data demonstrate that conventional analysis methods used in chemo-genetic profiling do not capture drug-induced death activation or drug-induced growth inhibition but rather an amalgam of these traits across four populations (*i.e.*, knockout and wild-type, in treated and untreated conditions). Notably, similar analytical strategies are used effectively in the context of gene essentiality profiling (55). A critical difference is that the relative fold change analysis used in gene essentiality profiling compares the dynamics of the knockout *versus* wild-type population with a static initial “T0” input population, which is used only to normalize for differences in starting population size. When applied in the context of chemo-genetic profiling, rather than gene essentiality, this analytical strategy confounds the interpretation, both from the perspective of death-activating *versus* growth-inhibiting drug effects, and regarding the “objective response” (*i.e.*, the degree to which the population is expanding or shrinking). For instance, a positive L2FC can be generated from a knockout population that is resistant to a drug, or from a knockout population that grows slowly in the absence of the drug without altering the drug response.

One unexpected finding from this study is that EGFR inhibitors do not typically activate high levels of cell death, even in the context of EGFR-mutant NSCLCs. Indeed, among the EGFR-mutant cell lines that are widely used, we identified only a single example, PC9 cells, which respond to EGFR inhibition with robust activation of apoptotic cell death. At least three plausible interpretations exist. First, because our study was performed under common tissue culture conditions, micro-environmental influences may alter the probability and levels of drug-induced cell death. Second, it remains possible that the clinically observed responses to EGFR inhibitors do not result from cell-autonomous forms of lethality, such as cell-intrinsic apoptosis. The methods of data collection and analysis that we used can accurately report all cell-autonomous types of apoptotic and non-apoptotic cell death; however, immune cell-mediated forms of cell death were not profiled. Finally, a third interpretation could be that EGFR inhibitor-induced lethality is indeed uncommon. PC9 cells may represent a “super responder” type clone. Future studies should aim to distinguish between these and potentially other possibilities.

Nonetheless, our data demonstrate that high levels of drug-induced cell death are possible following EGFR inhibition. The genetic dependencies that we identify should help to clarify the sources of response variation and should reveal rational strategies to improve the efficacy and durability of EGFR inhibitors.

## Experimental procedures

### Cells and reagents

NCI-H460 cells were acquired from the Green Laboratory (UMass Chan Medical School). A549, H1650, H1975, H1299, and PC9 cells were obtained from the American Type Culture Collection (ATCC). Cells were maintained at a low passage number, below 30, from the original vial and new vials were authenticated using STR profiling. A549 cells were grown in DMEM (Corning, 10–017-CV), and all other cells were grown in RPMI 1640 medium (Corning, 10-040-CV). All media was supplemented with 10% FBS (Peak Serum, PS-FB2) and penicillin–streptomycin (Corning, 30-002-CI).

SYTOX Green (S7020) was purchased from Thermo Fisher Scientific. Erlotinib hydrochloride salt was purchased from LC Laboratories (catalog no. E-4007), osimertinib was purchased from MedChemExpress (catalog no. HY-15772), buparlisib was purchased from MedChemExpress (catalog no. HY-70063), GSK690693 was purchased from SelleckChem (catalog no. S1113), and MK-2206 was purchased from SelleckChem (catalog no. S1078). Z-VAD-FMK was purchased from ApexBio (catalog no. A1902).

### FLICK-based analysis of drug response

The FLICK assay was conducted as described previously (47, 56). Briefly, cells were seeded at a density of 2000 cells per well in 96-well plates (Greiner Bio-One, 655,090) in 80 to 90 μl of media and incubated overnight. Cells were drugged with their respective doses of drug or vehicle in 2 to 5 μM of SYTOX Green media (Thermo Fisher Scientific, S7020) such that the final volume of each well was 100 μl. Dead cell fluorescence was measured with a Tecan Spark (ex: 503, em: 524), and gain was selected for each cell line as previously described (56). A T0 plate was lysed at the start of each assay by adding Triton X-100 (Thermo Fisher Scientific, BP151–100) to a final concentration of 0.15% in PBS and incubating at 37 °C for 3 h. At the end of the experiment, all plates were lysed using Triton X-100 a final concentration of 0.15% in PBS and incubated as before. The permeabilized plates provided the total number of cells at the start and end of the assay. From the total cell fluorescence and dead cell fluorescence, the live cell fluorescence can be inferred. This information allowed us to calculate RV, FV, LF, and GR (30). A custom MATLAB script was used to generate dose–response curves and LF kinetic curves as described previously (47).

### Gene expression analysis with RNAseq

To analyze drug-induced changes in gene expression, PC9 and H1650 cells were seeded at 200,000 cells per well in a six-well plate and incubated overnight. Cells were then treated with either 0.1% DMSO, or 10 μM erlotinib. T0 samples were collected at the time of drugging. After 36 h, the remaining samples were washed with 1 ml of PBS and removed from the plate with 500 μl of 1.5% trypsin. The cells were then spun down at 500*g* for 5 min, washed in PBS, and spun down again under the same conditions. The pellets were snap-frozen in liquid nitrogen and stored at −80 °C. RNA was extracted according to the manufacturer's instructions in the Qiagen RNeasy Mini Kit. Each sample had a concentration of at least 180 ng/μl, with 260/280 ratios ranging from 2.01 to 2.05 and 260/230 ratios ranging from one to 1.9. RNA was sent to Novogene for sequencing. Sequencing results were processed using the UMass Medical School Biocore's DolphinNext pipeline. DESeq2 in R computed log_2_(fold change) from the generated counts table (treated *versus* T0) and computed FDR-adjusted *p* values.

### Generation of PC9-Cas9 cell line

PC9-Cas9 cells were generated using a virus containing lenti-Cas9-Blast (Addgene, 52,962). Viral infection was performed by plating two million PC9 cells per well in a 12-well dish and adding virus, along with 8 μg/ml polybrene (Millipore, TR1003G). Cells were spun at 800*g* for 2 h at 37 °C, after which the viral media was removed and fresh media was added to allow the cells to recover overnight. The following day, cells were replated and allowed to recover further. Twenty-four hours later, cells were selected using 5 μg/ml blasticidin (ThermoFischer, R21001) for 5 days to generate a stable population. Cas9 expression was verified with a Western blot, and activity was evaluated using the Broad Institute's protocol for assaying Cas9 Activity with an EGFP reporter assay.

### Chemogenetic profiling of response to EGFR inhibition

A whole-genome CRISPR screen was performed using the TKOv3 two-vector system (40). PC9-Cas9 cells were transfected with the TKOv3 pooled library *via* spinfection. For each treatment condition (erlotinib, osimertinib, and DMSO), 45 × 10^6^ PC9-Cas9 cells were infected and divided into 12-well plates. Each well contained 1.5 × 10^6^ cells, 15 μl of virus, and 8 μg/ml polybrene (Millipore, TR1003G) in a total volume of 2 ml. The plates were centrifuged at 37 ˚C and 800*g* for 2 h. Following centrifugation, the media was replaced, and the cells were allowed to recover overnight. The cells were replated on 15 cm plates, incubated overnight, treated with 1 μg/ml puromycin (Corning, 61-385-RA) for 3 days, and then allowed to recover for 2 days. A total of 270 × 10^6^ cells were plated in total, with each experimental condition and T0 control having two replicates. On day 0, the treated conditions were drugged with 10 μM erlotinib, and the untreated conditions received 0.01% DMSO. The untreated conditions were passaged on day 1. On day 3, live cells from the treated and untreated conditions were collected and frozen.

DNA extraction, PCR and sequencing were performed as described previously (40). In brief, genomic DNA was isolated using the Wizard Genomic DNA Purification Kit (Promega, A1120) at a 10x scale. sgRNA sequences were extracted from each genome by PCR (forward: GAGGGCCTATTTCCCATGATTC, reverse: CAAACCCAGGGCTGCCTTGGAA). A second PCR reaction was used to attach barcodes used for multiplexing. Products from the second PCR reaction were gel-extracted (Qiagen, 28,704). Following library balancing, samples were sequenced on the Illumina NextSeq2000.

### Conventional analysis of chemogenetic profiles (L2FC)

Read quality was verified using FastQC, and non-variable regions were removed to isolate the guide sequences using the FASTX trimmer function. Reads were mapped to the TKOv3 library using Bowtie2, allowing a single mismatch. Counts tables were generated and the bottom 5% of guides by base mean were removed from the analysis. The L2FC was calculated at the guide level using DESeq2 in R. Guide-level scores were then colla'psed to gene-level by taking the median. The 142 non-targeting guides were randomly assigned to a “gene” in groups of 4. Gene-level fold changes were z-scored based on the distribution of L2FC scores for the non-targeting genes. An empirical *p* value was determined for each gene *via* 10,000 iterations, and this score was FDR-corrected using the Benjamini–Hochberg procedure.

### MEDUSA analysis

MEDUSA analysis for screen data was performed as previously described (20). Briefly, growth and death rates for PC9 cells, in the presence and absence of the drug, were determined using the FLICK assay and used to model population dynamics. With this model, we simulated all the ways a gene knockout might affect growth and drug-induced death rates. Using these simulated growth and death rates, the relative size of the treated and untreated populations can be determined. The conventional L2FC metric was then calculated. The drug-induced growth and death rates for each single-gene knockout was inferred from the observed L2FC, simulations, and the relative growth rate of the knockout in the absence of the drug. The MEDUSA-inferred guide level scores collapsed to the gene level by taking the mean, and then they were z-scored relative to the distribution of non-targeting genes. Empirical *p* values were determined *via* bootstrapping with 10,000 iterations and were FDR-corrected.

### Screen validation

Selected guides were cloned into the pX330-puro plasmid using the digestion-ligation protocol from the Zhang laboratory (available on the Zhang laboratory Addgene page). PC9 cells were plated at 300,000 cells per well in six-well dishes in complete medium. The following day, cells were transfected with 1.5 μg of sgRNA-puro constructs using the FuGENE HD Transfection Reagent (Promega, E2311) according to the manufacturer's instructions. The following day, cells were replated on 10 cm dishes. The next day, cells were treated with 1 μg/ml puromycin (Corning, 61-385-RA) and selected for 3 days. They were then washed twice with PBS and replaced with fresh media for recovery for 2 days. For single-cell cloning, selected cells were plated as single cells in 96-well plates, grown until confluent, and then expanded into 10 cm dishes. Afterward, genetic perturbations were validated *via* Western blot. sgRNA sequences cloned into px330 were:

PTEN - 5′ – ACCGCCAAATTTAATTGCAG – 3′

CASP3 – 5′ – TCTTGGCGAAATTCAAAGGA – 3′

APAF1 – 5′ – GTGAAGGTGGAGTACCACAG – 3′

PLCG1 – 5′ – ATAGCGATCAAAGTCCCGTG – 3′

PDK1 – 5′ – GTCACAAGAACTTCGACCAG – 3′

PIK3CA – 5′ – GAATAGGCAAGTCGAGGCAA – 3′

### Immunoblotting

Immunoblotting was performed using the LiCOR Odyssey CLx infrared scanner. PTEN (9188T), BIM (2933), Mcl-1 (5453S), and MYC (5605S) rabbit monoclonal antibodies were purchased from Cell Signaling Technologies. β-actin mouse monoclonal antibody was purchased from Sigma-Aldrich (A2228). Blots were blocked for 1 h at room temperature with 1:1 Intercept Blocking Buffer (LI-COR, 927-70003) and PBS. Blots were incubated at 4 °C for 20 h with primary antibody at a 1:1000 dilution, followed by 1 h incubation at room temperature, in 1:1 solution of blocking buffer and PBS-0.1% Tween (PBS-T). β-actin mouse primary antibody was incubated for 1 h at room temperature at a 1:15,000 dilution in 1:1 solution of blocking buffer and PBS-T. Blots were washed and incubated with LI-COR secondary antibodies at a 1:15,000 dilution (IRDye 680RD goat anti-mouse IgG, 926–68070; IRDye 800CW goat anti-rabbit IgG, 926-32211) for 1 h at room temperature in 1:1 solution of blocking buffer and PBS-T, washed 4 times for 5 minutes with PBS-T and once with PBS before imaging. Antibody specificity was validated by observing single bands at the expected size, which were lost upon genetic perturbation with targeted sgRNAs.

### Data analysis and statistics

Unless otherwise noted, data analysis was performed in MATLAB (version R2024a) using built-in functions. Bar graphs with individual data points were generated using GraphPad Prism (version 10.4.1). GRADE analysis was performed using the GRADE2.0 function (https://github.com/MJLee-Lab/GRADE2.0). GSEA analysis was performed using the GSEA 4.3.2 package. Pair-wise statistical comparisons were generated using a two-tailed two-sample *t* test. For conventional L2FC and MEDUSA analyses, *p* values were generated by bootstrapping with 10,000 iterations and were FDR-corrected using the Benjamini-Hochberg procedure.

## Data availability

Transcriptomic and chemo-genetic profiling data can be found in Supporting Information Tables 1 and 2 respectively, and as FASTQ files from the Gene Expression Omnibus (GSE323365, GSE323366). All other data are available in the main text or supporting information. Custom scripts used for curve fitting and analysis of drug response kinetics are deposited on GitHub (https://github.com/MJLee-Lab).

## Supporting information

This article contains supporting information.

## Conflict of interest

The authors declare that they do not have any conflicts of interest with the content of this article.

## References

[1] Zhong L., Li Y., Xiong L., Wang W., Wu M., Yuan T. (2021). Small molecules in targeted cancer therapy: advances, challenges, and future perspectives. Signal. Transduct. Target. Ther..

[2] Sabnis A.J., Bivona T.G. (2019). Principles of resistance to targeted cancer therapy: lessons from basic and translational cancer biology. Trends Mol. Med..

[3] Weinstein I.B., Begemann M., Zhou P., Han E.K., Sgambato A., Doki Y. (1997). Disorders in cell circuitry associated with multistage carcinogenesis: exploitable targets for cancer prevention and therapy. Clin. Cancer Res..

[4] Weinstein I.B., Joe A., Felsher D. (2008). Oncogene addiction. Cancer Res..

[5] Wakeling A.E., Barker A.J., Davies D.H., Brown D.S., Green L.R., Cartlidge S.A. (1996). Specific inhibition of epidermal growth factor receptor tyrosine kinase by 4-anilinoquinazolines. Breast Cancer Res. Treat..

[6] Fukuoka M., Yano S., Giaccone G., Tamura T., Nakagawa K., Douillard J.-Y. (2003). Multi-institutional randomized phase II trial of gefitinib for previously treated patients with advanced non–small-cell lung cancer. J. Clin. Oncol..

[7] Kris M.G., Natale R.B., Herbst R.S., Lynch J.T.J., Prager D., Belani C.P. (2003). Efficacy of gefitinib, an inhibitor of the epidermal growth factor receptor tyrosine kinase, in symptomatic patients with non–small cell lung cancer: a randomized trial. JAMA.

[8] Shepherd F.A., Pereira J.R., Ciuleanu T., Tan E.H., Hirsh V., Thongprasert S. (2005). Erlotinib in previously treated Non–small-cell lung cancer. N. Engl. J. Med..

[9] Khozin S., Blumenthal G.M., Jiang X., He K., Boyd K., Murgo A. (2014). U.S. food and drug administration approval summary: erlotinib for the first-line treatment of metastatic non-small cell lung cancer with epidermal growth factor receptor Exon 19 deletions or Exon 21 (L858R) substitution mutations. Oncologist.

[10] Kobayashi S., Boggon T.J., Dayaram T., Jänne P.A., Kocher O., Meyerson M. (2005). EGFR mutation and resistance of non–small-cell lung cancer to gefitinib. N. Engl. J. Med..

[11] Pao W., Miller V.A., Politi K.A., Riely G.J., Somwar R., Zakowski M.F. (2005). Acquired resistance of lung adenocarcinomas to gefitinib or erlotinib is associated with a second mutation in the EGFR kinase domain. PLoS Med..

[12] Yun C.-H., Mengwasser K.E., Toms A.V., Woo M.S., Greulich H., Wong K.-K. (2008). The T790M mutation in EGFR kinase causes drug resistance by increasing the affinity for ATP. Proc. Natl. Acad. Sci. U. S. A..

[13] Papadimitrakopoulou V.A., Wu Y.-L., Han J.-Y., Ahn M.-J., Ramalingam S.S., John T. (2018). LBA51 analysis of resistance mechanisms to osimertinib in patients with EGFR T790M advanced NSCLC from the AURA3 study. Ann. Oncol..

[14] Wee P., Wang Z. (2017). Epidermal growth factor receptor cell proliferation signaling pathways. Cancers.

[15] Akiyama T., Dass C.R., Choong P.F.M. (2009). Bim-targeted cancer therapy: a link between drug action and underlying molecular changes. Mol. Cancer Ther..

[16] Faber A.C., Li D., Song Y., Liang M.-C., Yeap B.Y., Bronson R.T. (2009). Differential induction of apoptosis in HER2 and EGFR addicted cancers following PI3K inhibition. Proc. Natl. Acad. Sci. U. S. A..

[17] Shan F., Shao Z., Jiang S., Cheng Z. (2016). Erlotinib induces the human non–small-cell lung cancer cells apoptosis via activating ROS-dependent JNK pathways. Cancer Med..

[18] Przybyla L., Gilbert L.A. (2022). A new era in functional genomics screens. Nat. Rev. Genet..

[19] Colic M., Hart T. (2019). Chemogenetic interactions in human cancer cells. Comput. Struct. Biotechnol. J..

[20] Honeywell M.E., Isidor M.S., Harper N.W., Fontana R.E., Birdsall G.A., Cruz-Gordillo P. (2024). Functional genomic screens with death rate analyses reveal mechanisms of drug action. Nat. Chem. Biol..

[21] Honeywell M.E., Lee M.J. (2025). MEDUSA for identifying death regulatory genes in chemo-genetic profiling data. J. Vis. Exp..

[22] Lo H.-W., Hung M.-C. (2006). Nuclear EGFR signalling network in cancers: linking EGFR pathway to cell cycle progression, nitric oxide pathway and patient survival. Br. J. Cancer.

[23] Gong Y., Somwar R., Politi K., Balak M., Chmielecki J., Jiang X. (2007). Induction of BIM is essential for apoptosis triggered by EGFR kinase inhibitors in mutant EGFR-dependent lung adenocarcinomas. Plos Med..

[24] Costa D.B., Halmos B., Kumar A., Schumer S.T., Huberman M.S., Boggon T.J. (2007). BIM mediates EGFR tyrosine kinase inhibitor-induced apoptosis in lung cancers with oncogenic EGFR mutations. PLoS Med..

[25] Cragg M.S., Kuroda J., Puthalakath H., Huang D.C.S., Strasser A. (2007). Gefitinib-induced killing of NSCLC cell lines expressing mutant EGFR requires BIM and can be enhanced by BH3 mimetics. PLoS Med..

[26] Matassa A.A., Carpenter L., Biden T.J., Humphries M.J., Reyland M.E. (2001). PKCδ is required for mitochondrial-dependent apoptosis in salivary epithelial cells. J. Biol. Chem..

[27] DeVries T.A., Neville M.C., Reyland M.E. (2002). Nuclear import of PKCδ is required for apoptosis: identification of a novel nuclear import sequence. EMBO J..

[28] Stephanou A., Latchman D.S. (2003). STAT-1: a novel regulator of apoptosis. Int. J. Exp. Pathol..

[29] Siddiquee K.A.Z., Turkson J. (2008). STAT3 as a target for inducing apoptosis in solid and hematological tumors. Cell Res..

[30] Hafner M., Niepel M., Chung M., Sorger P.K. (2016). Growth rate inhibition metrics correct for confounders in measuring sensitivity to cancer drugs. Nat. Methods.

[31] Sordella R., Bell D., Haber D.A., Settleman J. (2004). Gefitinib-sensitizing EGFR mutations in lung cancer activate anti-apoptotic pathways. Science.

[32] Ono M., Hirata A., Kometani T., Miyagawa M., Ueda S., Kinoshita H. (2004). Sensitivity to gefitinib (Iressa, ZD1839) in non-small cell lung cancer cell lines correlates with dependence on the epidermal growth factor (EGF) receptor/extracellular signal-regulated kinase 1/2 and EGF receptor/Akt pathway for proliferation. Mol. Cancer Ther..

[33] Schwartz H.R., Richards R., Fontana R.E., Joyce A.J., Honeywell M.E., Lee M.J. (2020). Drug GRADE: an integrated analysis of population growth and cell death reveals drug-specific and cancer subtype-specific response profiles. Cell Rep..

[34] Birdsall G.A., Lee M.J. (2025). Comprehensive analysis of drug response using the FLICK assay. J. Vis. Exp..

[35] Cruz-Gordillo P., Honeywell M.E., Harper N.W., Leete T., Lee M.J. (2020). ELP-dependent expression of MCL1 promotes resistance to EGFR inhibition in triple-negative breast cancer cells. Sci. Signal..

[36] Huether A., Höpfner M., Sutter A.P., Schuppan D., Scherübl H. (2005). Erlotinib induces cell cycle arrest and apoptosis in hepatocellular cancer cells and enhances chemosensitivity towards cytostatics. J. Hepatol..

[37] Tang Z.-H., Cao W.-X., Su M.-X., Chen X., Lu J.-J. (2017). Osimertinib induces autophagy and apoptosis via reactive oxygen species generation in non-small cell lung cancer cells. Toxicol. Appl. Pharmacol..

[38] Subramanian A., Tamayo P., Mootha V.K., Mukherjee S., Ebert B.L., Gillette M.A. (2005). Gene set enrichment analysis: a knowledge-based approach for interpreting genome-wide expression profiles. Proc. Natl. Acad. Sci. U. S. A..

[39] Kobayashi S., Shimamura T., Monti S., Steidl U., Hetherington C.J., Lowell A.M. (2006). Transcriptional profiling identifies cyclin D1 as a critical downstream effector of mutant epidermal growth factor receptor signaling. Cancer Res..

[40] Hart T., Tong A.H.Y., Chan K., Leeuwen J.V., Seetharaman A., Aregger M. (2017). Evaluation and design of genome-wide CRISPR/SpCas9 knockout screens. G3.

[41] Hanna R.E., Doench J.G. (2020). Design and analysis of CRISPR–cas experiments. Nat. Biotechnol..

[42] Bao Q., Shi Y. (2007). Apoptosome: a platform for the activation of initiator caspases. Cell Death Differ..

[43] Anvar N.E., Lin C., Ma X., Wilson L.L., Steger R., Sangree A.K. (2024). Efficient gene knockout and genetic interaction screening using the in4mer CRISPR/Cas12a multiplex knockout platform. Nat. Commun..

[44] Parrish P.C.R., Thomas J.D., Gabel A.M., Kamlapurkar S., Bradley R.K., Berger A.H. (2021). Discovery of synthetic lethal and tumor suppressor paralog pairs in the human genome. Cell Rep..

[45] Stambolic V., Suzuki A., de la Pompa J.L., Brothers G.M., Mirtsos C., Sasaki T. (1998). Negative regulation of PKB/Akt-Dependent cell survival by the tumor suppressor PTEN. Cell.

[46] Maehama T., Dixon J.E. (1998). The tumor suppressor, PTEN/MMAC1, dephosphorylates the lipid second messenger, phosphatidylinositol 3,4,5-Trisphosphate. J. Biol. Chem..

[47] Richards R., Schwartz H.R., Honeywell M.E., Stewart M.S., Cruz-Gordillo P., Joyce A.J. (2020). Drug antagonism and single-agent dominance result from differences in death kinetics. Nat. Chem. Biol..

[48] Meyer C.T., Wooten D.J., Lopez C.F., Quaranta V. (2020). Charting the fragmented landscape of drug synergy. Trends Pharmacol. Sci..

[49] Kwon E.-J., Cha H.-J., Lee H. (2024). Systematic omics analysis identifies CCR6 as a therapeutic target to overcome cancer resistance to EGFR inhibitors. iScience.

[50] Guler G.D., Tindell C.A., Pitti R., Wilson C., Nichols K., Cheung T.K. (2017). Repression of stress-induced LINE-1 expression protects cancer cell subpopulations from lethal drug exposure. Cancer Cell.

[51] Giaever G., Chu A.M., Ni L., Connelly C., Riles L., Véronneau S. (2002). Functional profiling of the Saccharomyces cerevisiae genome. Nature.

[52] Zeng H., Castillo-Cabrera J., Manser M., Lu B., Yang Z., Strande V. (2019). Genome-wide CRISPR screening reveals genetic modifiers of mutant EGFR dependence in human NSCLC. eLife.

[53] Lee J., Choi A., Cho S., Jun Y., Na D., Lee A. (2021). Genome-scale CRISPR screening identifies cell cycle and protein ubiquitination processes as druggable targets for erlotinib-resistant lung cancer. Mol. Oncol..

[54] Pfeifer M., Brammeld J.S., Price S., Pilling J., Bhavsar D., Farcas A. (2024). Genome-wide CRISPR screens identify the YAP/TEAD axis as a driver of persister cells in EGFR mutant lung cancer. Commun. Biol..

[55] Tsherniak A., Vazquez F., Montgomery P.G., Weir B.A., Kryukov G., Cowley G.S. (2017). Defining a cancer dependency map. Cell.

[56] Richards R., Honeywell M.E., Lee M.J. (2021). FLICK: an optimized plate reader-based assay to infer cell death kinetics. STAR Protoc..

