# Supplementary material for: Genome-wide profiling identifies the genetic dependencies of cell death following EGFR inhibition
Source: J Biol Chem. 2026 Apr 2;302(5):111414. doi: 10.1016/j.jbc.2026.111414 (PMC13133947; doi:10.1016/j.jbc.2026.111414)
Supplement: Figures S1–S7 [file mmc1.pdf]

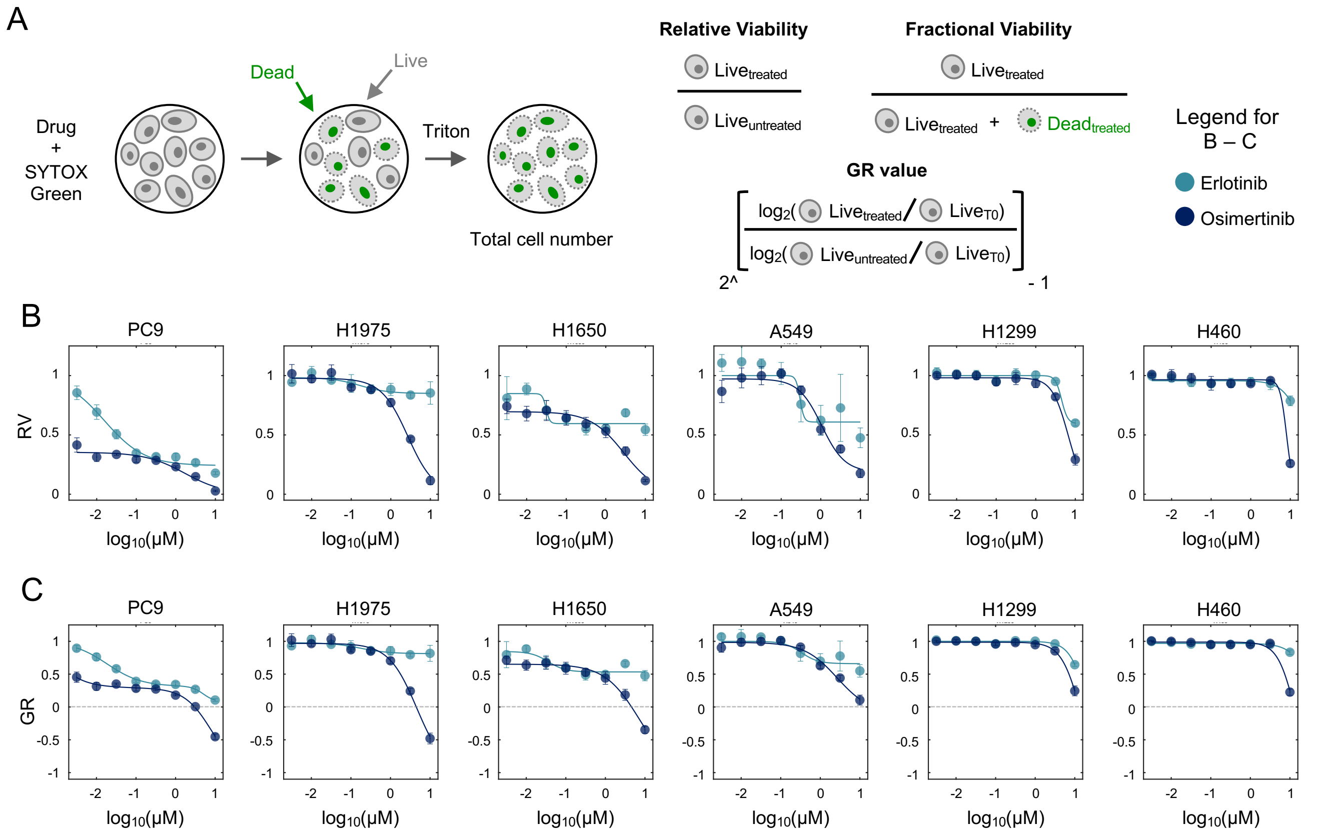

Supplementary Figure 1. Profiling the sensitivity of EGFR inhibitors in NSCLC cells. (**A**) Schematic of the FLICK assay and equations for calculating relative viability (RV), fractional viability (FV), and normalized growth rate inhibition (GR) values. (**B**) RV of PC9, H1975, H1650, A649, H1299, and H460, evaluated after 72 hours of treatment with erlotinib or osimertinib. RV values calculated as described in (A). (**C**) GR of PC9, H1975, H1650, A649, H1299, and H460, evaluated after 72 hours of treatment with erlotinib or osimertinib. GR values calculated as described in (A). For all panels with error bars, data are the mean ± S.D. for *n* = 3 independent biological replicates.

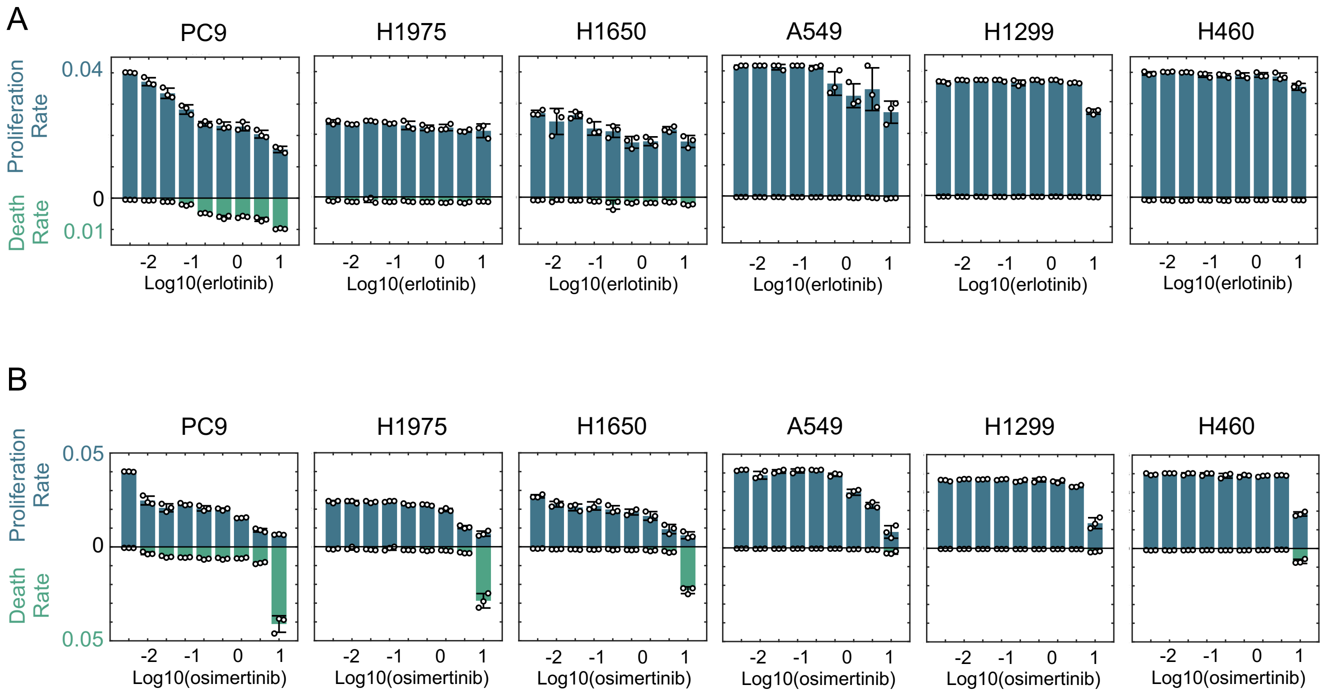

Supplementary Figure 2. Erlotinib uniquely increases death rate in PC9 cells while high osimertinib doses are lethal regardless of mutation status. (**A**) Proliferation rate (doublings/hour) and death rate (LF/hour) for cell lines treated with an 8-point dose titration of erlotinib. PC9, H1975, and H1650 are EGFR mutants while the remaining cell lines are EGFR wild type. Values calculated from GRADE analysis. (**B**) As in (A) but for cells treated with osimertinib. For all panels with error bars, data are the mean ± S.D. for *n* = 3 independent biological replicates.

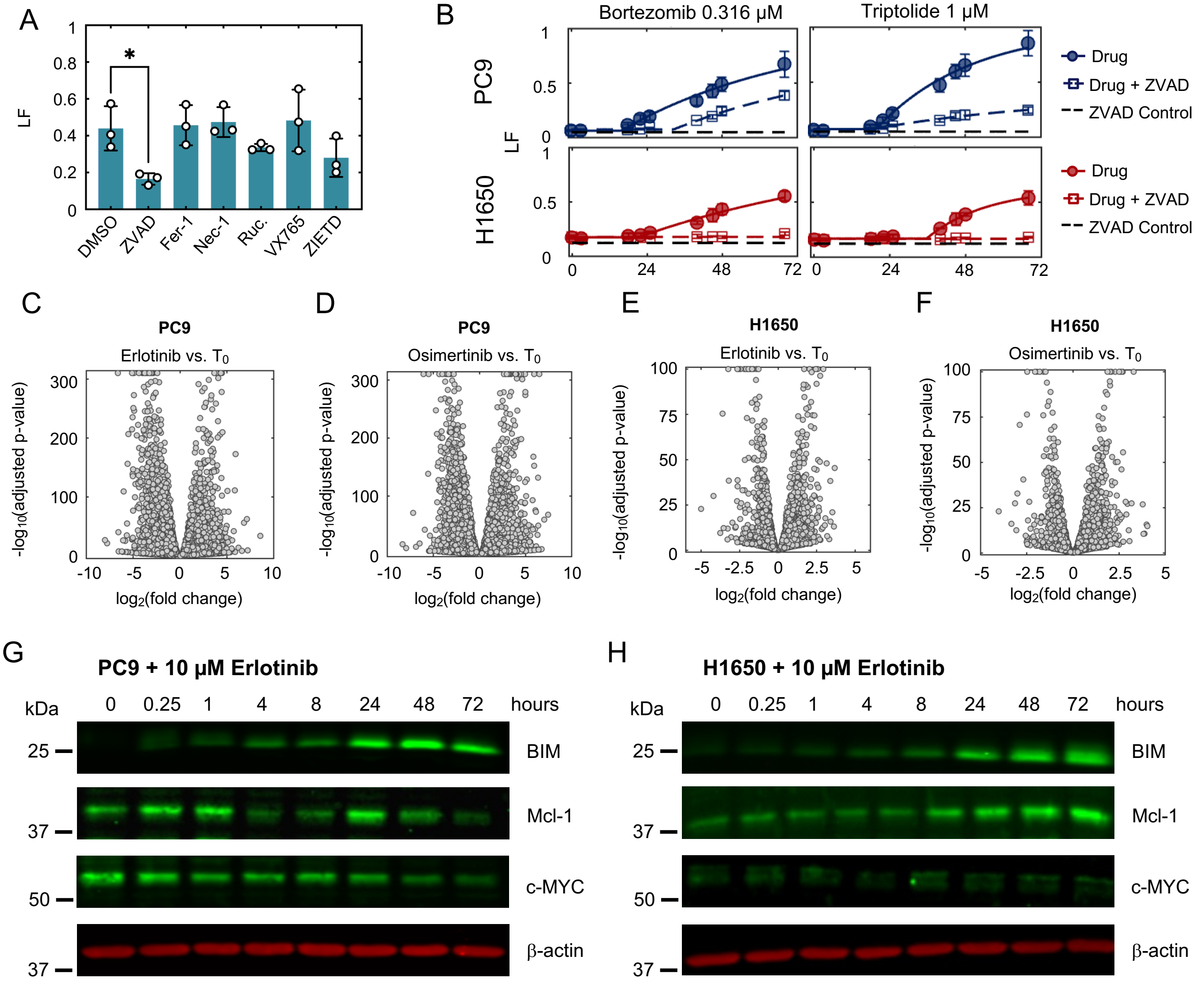

Supplementary Figure 3. RNA-seq reveals gene expression changes following EGFR inhibitor treatment. (**A**) 72-hour lethal fraction (LF) for PC9 cells exposed to 10 μM erlotinib presence of different cell death inhibitors. 50 μM z-VAD used to inhibit intrinsic apoptosis, 10 μM Ferrostatin-1 to inhibit ferroptosis, 10 μM Necrostatin-1 to inhibit necroptosis, 1 μM Rucaparib to inhibit parthanatos, 50 μM VX765 to inhibit pyroptosis, and 31.6 μM ZIETD to inhibit extrinsic apoptosis. *$p < 0.01$ using a two-sided *t*-test. (**B**) LF kinetics measured using the FLICK assay following exposure to Bortezomib or Triptolide, in the presence or absence of ZVAD. **(C)** RNA-seq for expression changes in PC9 cells treated with 10 μM erlotinib for 36 hours. Volcano plot showing the -log$_{10}$FDR p-values and log$_2$-fold change of the drug treated cells compared to T_0 (log$_2$(T_{36}/T_0)). Data is the mean of two biological replicates. (**D**) As in (C) for PC9 cells treated with 0.1 μM osimertinib. (**E**) As in (C) for H1650 cells treated with 10 μM erlotinib. (**F**) As in (C) for H1650 cells treated with 0.1 μM osimertinib. **(G-H)** Protein levels for key death regulatory proteins following Erlotinib exposure. BIM is induce at the mRNA level; MCL1 and MYC are not changed at the mRNA level. Data are representative of 3 biological replicates. (G) PC9 cells. (H). H1650 cells. For all panels with error bars, data are the mean ± S.D. for n = 3 independent biological replicates.

Porto, et al., Sup. Figure 4

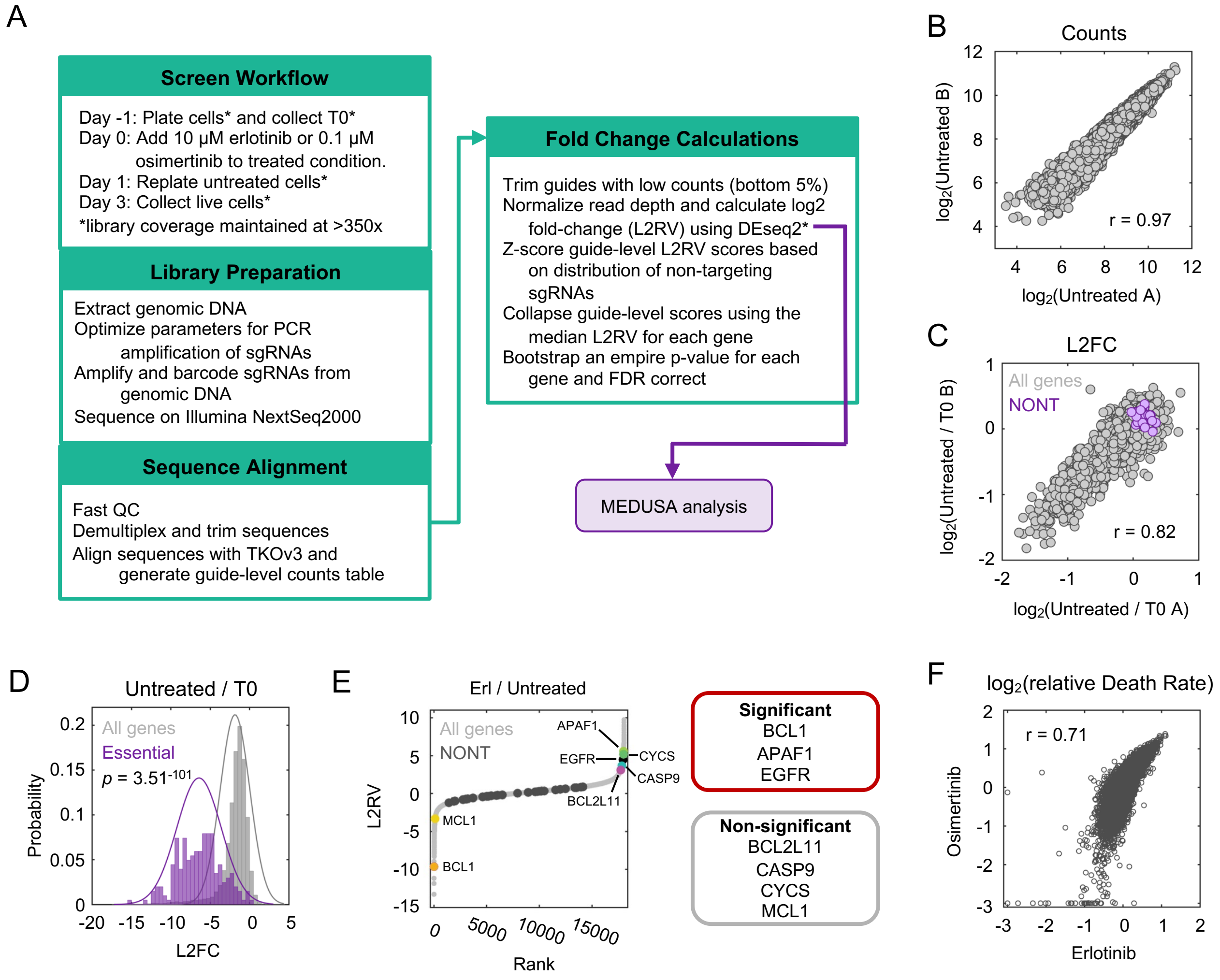

Supplementary Figure 4. Chemo-genetic screening analysis strategy and quality control. (**A**) Analysis pipeline for calculating L2RV and MEDUSA analysis from chemo-genetic screen. (**B**) Correlation between counts for two replicates of untreated sample. Representative of the counts correlation between replicates for each of the screening conditions. Pearson Correlation Coefficient (r) shown. (**C**) Correlation between gene-level $\log_2$relative viability (L2RV) values for untreated samples compared to T_0 (Untreated/T_0) with two replicates. Non-targeting (NONT) sgRNAs shown in purple, all other genes shown in light gray. Representative of the L2RV correlation between replicates for each of the screening conditions. Pearson Correlation Coefficient (r) shown. (**D**) Evaluation of screen's ability to identify essential genes. Distribution of gene-level L2FC scores for core essential genes (Essential) vs. all genes in untreated vs. T0 comparison. Two-sided KS test p-value shown. (**E**) Conventional L2FC analysis for screen with erlotinib. Non-targeting (NONT) sgRNAs shown in dark gray, and key hits from MEDUSA analysis are highlighted. (**F**) Gene-level correlation between $\log_2$(relative Death Rate) from MEDUSA analysis for erlotinib and osimertinib conditions. Pearson Correlation Coefficient (r) shown.

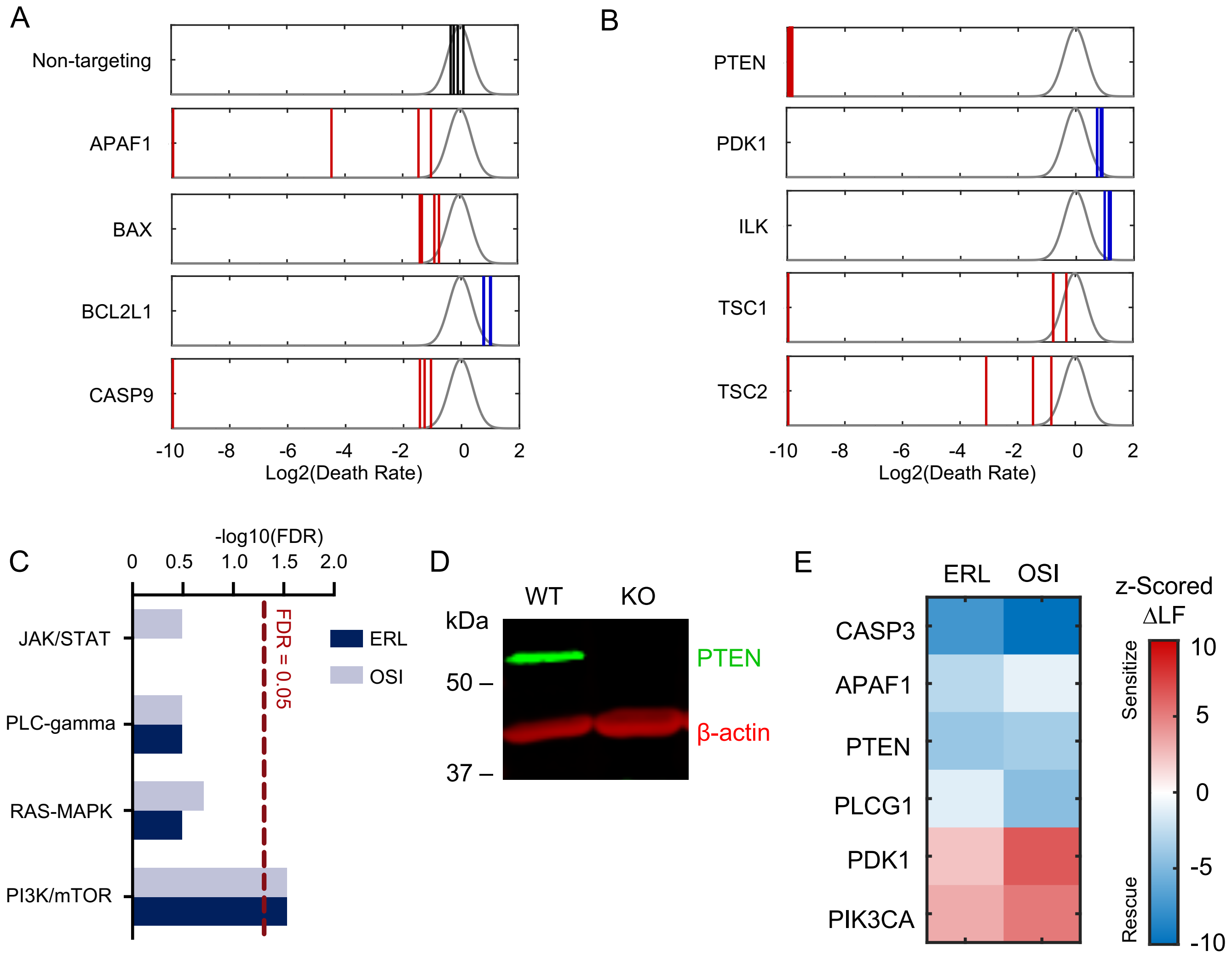

Supplementary Figure 5. Validation of results from chemo-genetic profiling. (A) Examples of non-targeting and apoptotic regulatory genes, which are negative and positive controls, respectively. Positive regulators of cell death are red; negative regulators of cell death are blue. Distribution shown in grey represents all sgRNAs. **(B)** As in panel (A) but showing examples of death regulatory genes in the PI3K signaling pathway. For PTEN, all 4 sgRNAs fall in the same region. **(C)** Pathway-level enrichment for genes that regulate the lethality of erlotinib (ERL) or osimertinib (OSI). Enrichment was calculated using the Fisher's Exact test. Data are the FDR-adjusted p-values. **(D)** Western blot for PTEN knockout clone validation in PC9 cells. See also Figure 4C. **(E)** Validation of MEDUSA-based inferences using transient expression of a plasmid encoding Cas9 and an sgRNA targeting the listed gene. Cells were exposed to 10 μM Erlotinib (ERL) or 0.1 μM Osimertinib (OSI) for 72 hours, and the drug-induced lethal fraction (LF) was calculated using the FLICK assay. Based on the MEDUSA-inferred death rates in the chemo-genetic profiling experiment, knocking out CASP3, APAF1, PTEN, and PLCG1 were predicted to cause decreased cell death ("Rescue"), whereas knocking out PDK1 (PDPK1 gene) or PIK3CA were predicted to cause an increased level of cell death ("Sensitize"). Data are based on means and standard deviations from 3 independent biological replicate experiments.

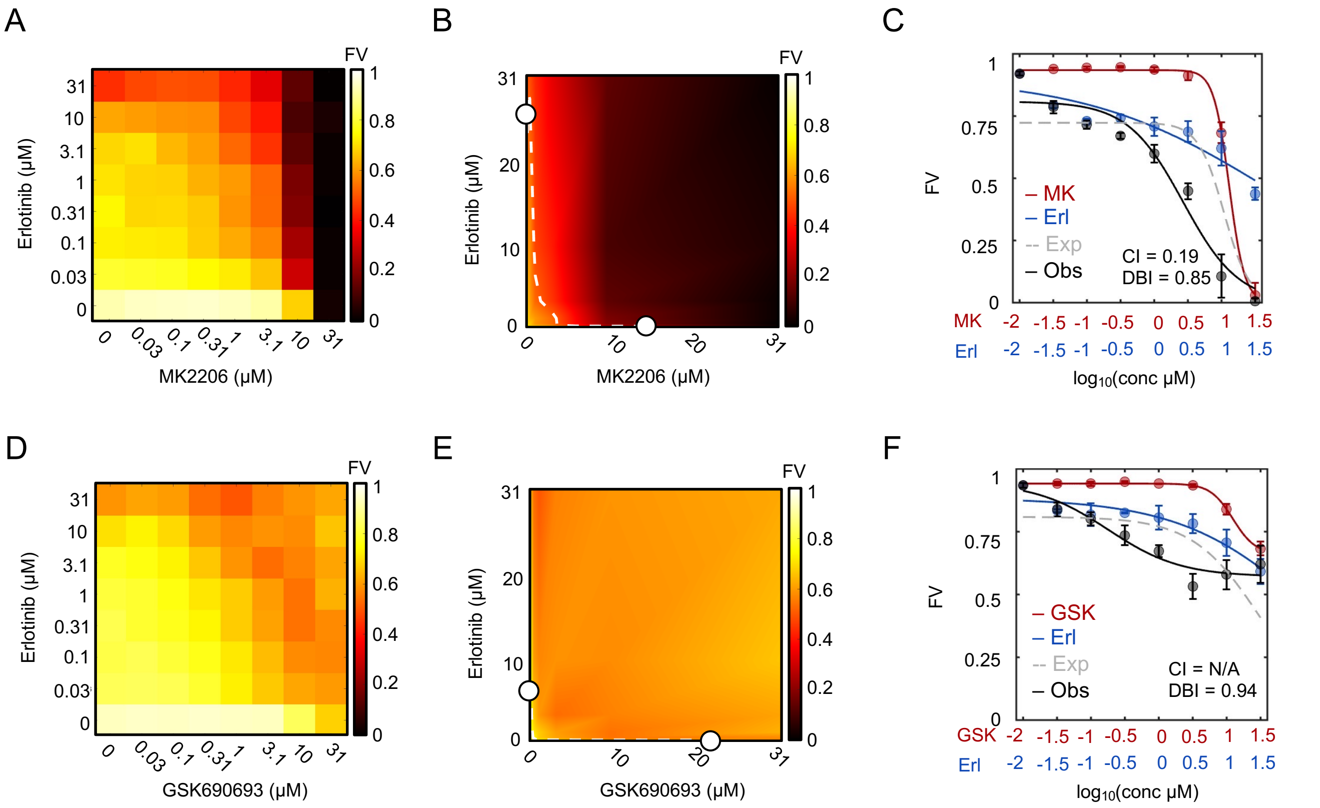

Supplementary Figure 6. Synergistic drug-drug interactions between EGFR inhibitors and AKT inhibitors in PC9 cells. (**A-C**) Dose titration of erlotinib and AKT Inhibitor, MK2206 in PC9 cells. **(A)** Heatmap is scaled by the mean fractional viability of 3 biological replicates following 72 hours of drug treatment. (**B**) Isobologram analysis for the data in (B). The dashed line represents the erlotinib and buparlisib combinations that result in 50% response (50% isobol). White dots show the single drug IC50 doses. (**C**) Dose curve for erlotinib and MK2206 at fixed ratio dosing. The expected dose curve in the case of additivity is shown in addition to the observed combination. The Chou-Talalay Combination Index (CI) and the Deviation from Bliss Independence (DBI) are shown. (**D-F**) Dose titration of erlotinib and AKT Inhibitor, GSK690693 in PC9 cells. **(D)** As in panel (A) but for combinations of erlotinib-GSK690693. (**E**) As in panel (B) but for combinations of erlotinib-GSK690693. The dashed line represents the erlotinib and buparlisib combinations that result in 70% response (70% isobol), because no conditions reached an IC50 response. (**F**) As in panel (C) but for combinations of erlotinib-GSK690693.

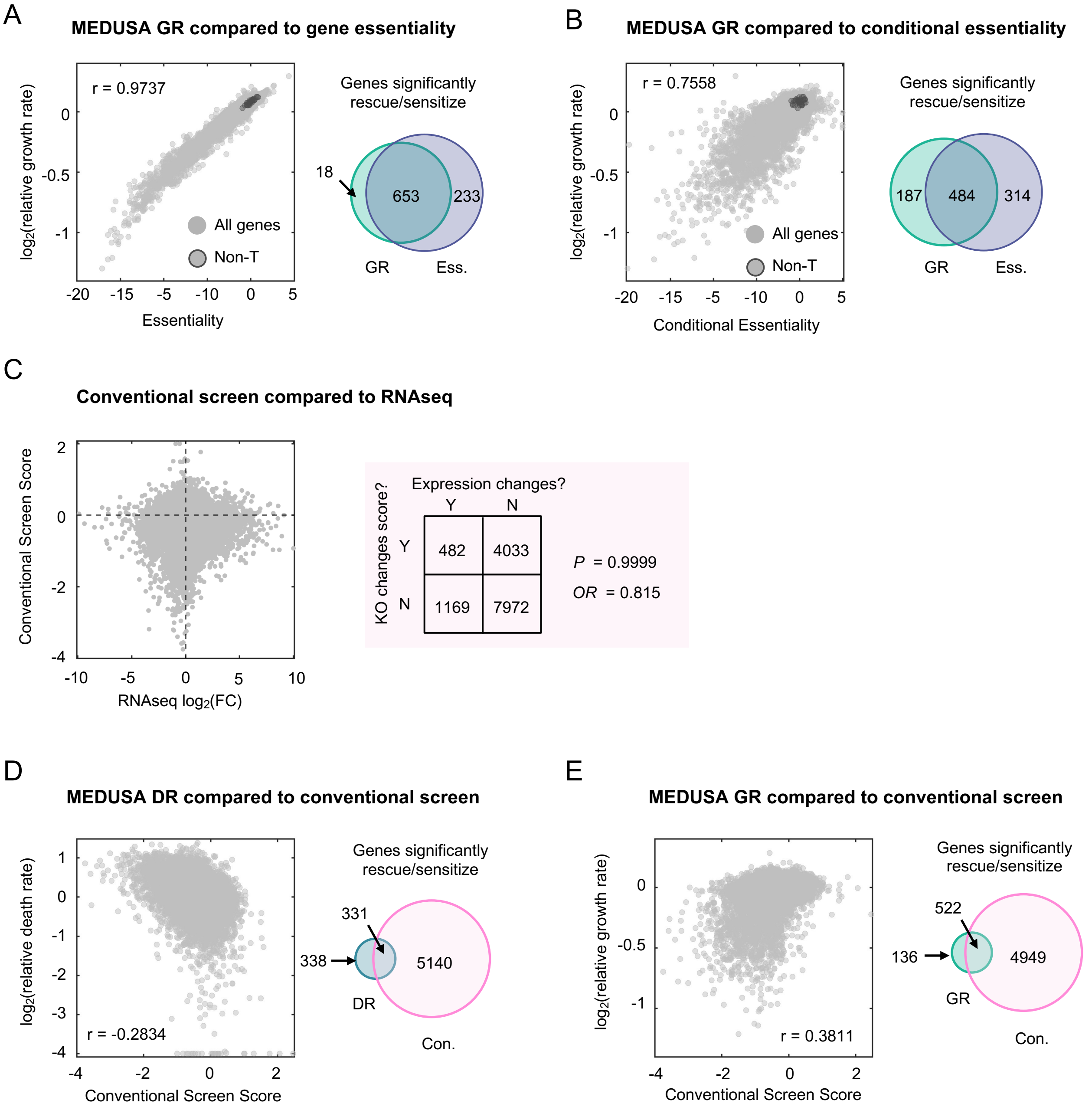

Supplementary Figure 7. MEDUSA GR captures gene essentiality but MEDUSA metrics still provide distinct insights compared to conventional approaches. (**A**-**B**) Comparison of the MEDUSA GR metric to conventional metrics used in functional genomics and/or chemo-genetic profiling. (A) Comparison between MEDUSA GR and gene essentiality. (B) Comparison between MEDUSA GR and conditional essentiality. Pearson Correlation Coefficients (r) shown. Venn diagrams show the relative number and relationship between “hits” recovered by each analysis strategy. (**C**) Comparison of conventional screen score and drug-induced gene expression changes. Odds Ratio (OR) and p-value based on Fisher’s Exact Test to determine the relationship between gene expression changes and conventional screen score. (**D**-**E**) Comparison of the MEDUSA metrics to scores from a conventionally conducted screen. (D) Comparison between MEDUSA DR and conventional screen score. (E) Comparison between MEDUSA GR conventional screen score. Pearson Correlation Coefficients (r) shown. Venn diagrams show the relative number and relationship between “hits” recovered by each analysis strategy.
